# Observations and Experiments on Cupping-Glasses and Pressure in Poisoned Wounds

**Published:** 1828-05

**Authors:** 


Art. III.—*Observations and Experiments on the Efficacy and Modus Operandi of Cupping-Glasses, in preventing and arresting the effects of Poisoned Wounds. By* Caspar Wistar Pennock, M. D. *Philadelphia*, 1828. *pp.* 20.

The experiments and conclusions of Dr. Barry, on the influence of cupping glasses, in preventing the deleterious effects of poisons introduced into wounds, are now so generally known to our readers, that we consider it unnecessary to state them in our notice of this little work. Dr. Pennock's design, was to ascertain their correctness. For this purpose, he employed concentrated hydrocyanic acid, strichnine, upas tieute, and arsenious acid. His experiments were performed on rabbits, and seem to have been conducted with the requisite skill and circumspection, in the presence of several medical gentlemen. The following are his conclusions:

*First.* The usual effects of poisoned wounds cannot take place during the absence of the atmospheric pressure procured by the application of cupping-glasses.

*Second.* Such application does not arrest the deleterious action of the poison by withdrawing it from the exposed surface; on the contrary, the fatal effects are wholly prevented, though not a particle of the substance employed has been abstracted. In proof of this, if a poison, in powder, (strychnine or arsenic for instance,) be conveyed by a tube through a narrow wound, in an oblique direction under the integuments, to some distance from the opening by which it is introduced, and there deposited—and under these circumstances the glass be applied over this spot, where the skin is sound and unbroken, the wound being without the bounds of the glass, none of the poisonous substance will be removed, and yet no indication of its action will be presented during the time of the application of the glass.

*Third.* The constitutional symptoms, such as tetanic convulsions, etc. are arrested by the establishment of a vacuum on the poisoned surface; then by removing the poison by an incision through the integuments, the life of the animal is preserved.

*Fourth.* When the cupping-glass is applied over the opening made in the integuments for the purpose of introducing the tube containing the poison, and this is deposited under the skin beyond the circumference of the glass, none of the effects are manifested during the continuance of the vacuum, but as soon as the cup is removed, the action of the deleterious article commences.

*Fifth.* If during the application of the cupping glass, placed as just stated, an incision be made between its edge and the place at which the poison has been lodged, death will ensue as speedily as though the atmospheric pressure had not been removed.

*Sixth.* If after the application of a glass for a given time, to the sound skin over the spot where the poison has been deposited, the glass be removed, death will then ensue as soon as if no such application had been made.

This last position is entirely at variance with the observations of Dr. Barry. He expressly says, that "after the glass had been taken off, the animal continued for one or two hours to carry imbedded in his cellular tissue a dose which would infallibly have destroyed him in a few minutes, had the cupping-glasses not been previously applied.

I have repeatedly observed, however, that if the animal was abandoned to his fate after the glass had been removed, after an application of it for an hour or more, that death took place as soon afterwards as it ordinarily did when no vacuum had been formed."—pp. 6—7.

Not satisfied with Dr. Barry's explanation of the *modus operandi* of cupping-glasses in the production of these effects, Dr. Pennock sought for some other. The following are his words:

"Agreeably to Dr. Barry's view, external absorption is not a vital function, but it, as well as the circulation in the absorbing vessels, is a physical effect caused by atmospheric pressure, the fluids passing through the absorbents precisely as a liquid is forced into, and rises into a Torricellian vacuum.

Dr. Barry, in support of his opinions, appeals to the series of experiments: the truth of the results of which, as stated by him, I have had the pleasure to confirm. His view, however, of the modus operandi of the cupping-glasses in preventing the effects of external poisons are, I am induced to believe, entirely erroneous.

The results of some experiments which I instituted with a view of testing the principle, whether their efficacy *depends merely on the abstraction of atmospheric pressure*,, have convinced me that such is not the fact.

It is true that the exhaustion of air from within the bounds of the glass, relieves that portion of the body inclosed within its periphery from the usual atmospheric pressure: but the parts *beneath* the *circumference* of the cup, suffer an increase of pressure proportionate to the diameter of the glass. Thus the glass which I used, being one inch and a quarter in diameter, the animal suffered an increase of pressure on the part just indicated of at least fifteen pounds." p. 16.

To this *pressure* Dr. P. was led to ascribe the efficacy of cupping-glasses, and proceeded to verify his theory by experiment. He has given us the details of three, which he regards as conclusively in the affirmative. It is not, then, the *diminution* of pressure within the circumference of the glass, when exhausted of air, but the necessary *increase* of pressure under that circumference, from the weight of the air upon the glass, which prevents the constitutional effects of the poison. This pressure, he remarks, may arrest the functions of both the vascular and nervous systems of the part, and thereby prevent a constitutional disturbance, whether it results from absorption or sympathy. Admitting the fact, which we see nothing to justify us in denying, it follows, that pressure to or about the poisoned wound, by any other instrument or means than the cupping-glass, provided it be equal in degree, will be equal in effect; a practical fact of importance, seeing that in the country, cupping glasses and even the skill necessary to use them with efficacy, are often wanting. Pressure over the part to the amount of 15 or 20 pounds to the square inch being the *desideratum*, it may be made in any manner that is at the time most convenient. It should be continued for hours or even days.

From the great efficacy of cupping, and of pressure by other means, in arresting tetanic symptoms from strichnine, Dr. P. is inclined to recommend the same application in traumatic tetanus, or in wounds which threaten to occasion that desperate malady. The suggestion seems plausible, and we hope that such of our readers as may have an opportunity, (the number we trust will be few) will think it worthy of being tested by experiment.

